# Analysis of Ionospheric Disturbances Caused by the 2018 Bering Sea Meteor Explosion Based on GPS Observations

**DOI:** 10.3390/s20113201

**Published:** 2020-06-04

**Authors:** Yiyong Luo, Yibin Yao, Lulu Shan

**Affiliations:** 1 School of Geodesy and Geomatics, Wuhan University, Wuhan 430079, China; luoyiyong@whu.edu.cn (Y.L.); llshan@whu.edu.cn (L.S.); 2 Faculty of Geomatics, East China University of Technology, Nanchang 330013, China

**Keywords:** meteor explosion, TEC, TID, characteristics of TID

## Abstract

The Bering Sea meteor explosion that occurred on 18 December 2018 provides a good opportunity to study the ionospheric disturbances caused by meteor explosions. Total electron content (TEC) is the core parameter of ionospheric analysis. TEC and its changes can be accurately estimated based on the Global Positioning System (GPS). TID is detected in time and frequency domain based on power spectrum and Butterworth filtering method. By analyzing the waveform, period, wavelength, propagation speed and space-time distribution of TID, the location of the TID source is determined, and the process of TID formation and propagation is understood. The TID caused by meteor explosions has significant anisotropy characteristic. Two types of TID were found. For the first type, the average horizontal propagation velocity is 250.22 ± 5.98 m/s, the wavelength is ~135–240 km, the average period is about 12 min, and the propagation distance is less than 1400 km. About 8 min after the meteor explosion, the first type of TID source formed and propagated radially at the velocity of 250.22 ± 5.98 m/s. For the second type, the propagation velocity is ~434.02 m/s. According to the waveform, period, wavelength and propagation velocity of the TID, it is diagnosed to be the midscale traveling ionospheric disturbances (MSTID). Based on the characteristics of TID, we infer that the TID is excited by the gravity waves generated by the meteor explosion, which is in accordance with the propagation law of gravity waves in the ionosphere. And it is estimated that the average velocity of the up-going gravity waves is about 464.58 m/s. A simple model was established to explain the formation and the propagation of this TID, and to verify the characteristics of the TID propagation caused by nuclear explosion, earthquake, tsunami, and Chelyabinsk meteorite blast. It is estimated that the position of the TID source is consistent with the meteor explosion point, which further indicates that the TID is caused by the meteor explosion and propagates radially.

## 1. Introduction

Images taken by the Japanese Sunflower 8 satellite and US Terra satellite of the meteoroid crossing the atmosphere confirmed that a huge meteor caused an explosion in the Bering Sea near the Kamchatka Peninsula in Russia (called the Bering Sea meteor explosion) on 18 December 2018 at 23:48:20 (UT). According to NASA’s estimates, the meteor entered the atmosphere at a velocity of approximately 32 km/s and exploded at 56.9° N, 172.4° E, and 25.6 km from the ground. The meteor explosion released approximately 173,000 tons of TNT equivalent, which is more than 10 times that of the Hiroshima atomic bomb explosion in in 1945 during World War II. It was the second largest meteor explosion since 1908, and its energy is second only to the 2013 Chelyabinsk meteor explosion (https://cneos.jpl.nasa.gov/fireballs/). The hypersonic bolide generated powerful shock waves while acoustic perturbations in the atmosphere led to the upward acoustic and gravity waves propagating into the ionosphere, which causes ionospheric disturbances. The tiny soot particles deposited by meteors in the upper atmosphere and ionosphere can lead to microphysical local ionization processes [1,2]. They also generate infrasonic perturbations that can interact with the neutral atmosphere generating acoustic and gravity waves that propagate into the ionosphere as measured by trans ionospheric Global Positioning System (GPS) and infrasound sensors. Due to the scarcity of the detected large meteor explosions, there are few examples that can be used to study the ionospheric disturbances caused by meteor explosions. At present, there are only some studies about the ionospheric disturbance caused by Chelyabinsk meteor explosion in 2013. Recent studies show that meteor explosions can produce sound waves, gravity waves, seismic waves, etc., which can propagate into the ionosphere, causing ionospheric disturbances. Because the mechanism of ionosphere disturbance caused by meteor explosion is not well understood, it is necessary to further study the characteristics of the ionosphere disturbance. The Bering Sea meteor explosion event provides a good opportunity to further study the phenomenon of meteor explosion-induced ionospheric disturbances.

Currently, GPS, ionospheric ionosonde, and radar observation are widely used in monitoring ionospheric disturbances caused by earthquakes, tsunamis, volcanoes, and nuclear explosions. Increasing evidences have showed that earthquakes, tsunamis, volcanoes, nuclear explosions, and rocket launches, etc. can produce acoustic waves, gravity waves, and seismic waves, which further cause travelling ionospheric disturbances (TID). Using the above detection techniques, changes of the TID are detected by analyzing the ionospheric parameters, including total electron content, and electron density, etc. [3,4,5,6,7,8]. Studies on large meteor explosions in the lower atmosphere are rare, and the current studies mainly focus on the ionospheric disturbances caused by the 2013 Chelyabinsk meteor explosion. The ionospheric disturbances caused by the Chelyabinsk meteor explosion were observed using the radar data of ARTI station located 200 km from the explosion point to determine the propagation velocity of the three ionospheric disturbances which velocities are 250, 400, and 800 m/s, respectively [9]. Berngardt used radar and ionosonde data to analyze the TID characteristics of a meteor falling and exploding within 25 min, and the TID patterns induced by sound waves and seismic waves were analyzed [10]. Pradipta et al. used ionosonde data to analyze the ionospheric parameter variation caused by the meteor explosion and determined the ionospheric disturbance velocity to be 171 m/s [11]. The close-range ionospheric disturbance caused by the meteor explosion was detected by analyzing the changes in total electron content (TEC) at the ARTU station and then was compared the data with the ionospheric disturbance caused by the nuclear explosion [12,13]. Ding et al. (2016) used the GPS data from the Crustal Movement Observation Network of China and detected ionospheric disturbances with a propagation velocity of 410 m/s and located 2500–3000 km from the explosion point [14]. Yang et al. used the Japanese regional GPS network near the meteor explosion point, with the US regional GPS network to observe the ionospheric disturbances caused by three meteor explosions at speeds of 362, 733 and 862 m/s, respectively [15]. Perevalova et al. analyzed three ionospheric disturbance modes using 10 GPS stations located near the explosion point, with a perturbation propagation velocity of 250–660 m/s and estimated the location of the ionospheric disturbance source [16]. The above analysis confirms that the GPS observations can be used to accurately analyze the ionospheric disturbance characteristics. Berngardt et al. studied the azimuthal characteristics of ionospheric and the seismic effects of the meteorite ‘Chelyabinsk’, based on the data from the network of GPS receivers, coherent decameter radar EKB, and network of seismic stations, located near the meteorite fall trajectory [17]. At present, there are few studies about ionosphere disturbance caused by other meteor explosions and there are currently no relevant studies on the ionospheric disturbances caused by the Bering Sea meteor explosion.

TEC is the core parameter that describes the characteristics of the ionosphere. The TEC information obtained by GPS observations can be used to detect the ionospheric disturbance. In this paper, GPS dual-frequency observations from one near-filed GPS station and 20 far-field GPS stations are used to calculate TEC between the station and visible satellite, which is then used to investigatethe ionospheric disturbance induced by the Bering Sea meteor explosion. By analyzing the spectrum of TEC time series, the characteristics of TID in time and frequency domain are detected. Then, the waveform, propagation velocity, wavelength and spatial-temporal distribution characteristics of TID are analyzed based on Butterworth band pass filter. Finally, the location of TID source is estimated, and a simple TID model is established to explain the formation and propagation mechanism of TID. The contents of this paper are organized as follows: The research data and processing methods are introduced in Section 2; analysis of ionospheric disturbance characteristics in the vicinity of meteor explosion at AC60 station in Section 3; the characteristics of long-distance TID are analyzed using GPS observations in Section 4; estimation of TID propagation velocity and disturbance source location in Section 5; discussion and conclusions follow in Section 6.

## 2. Data and Methodology

### 2.1. Data Introduction

**Figure 1:**
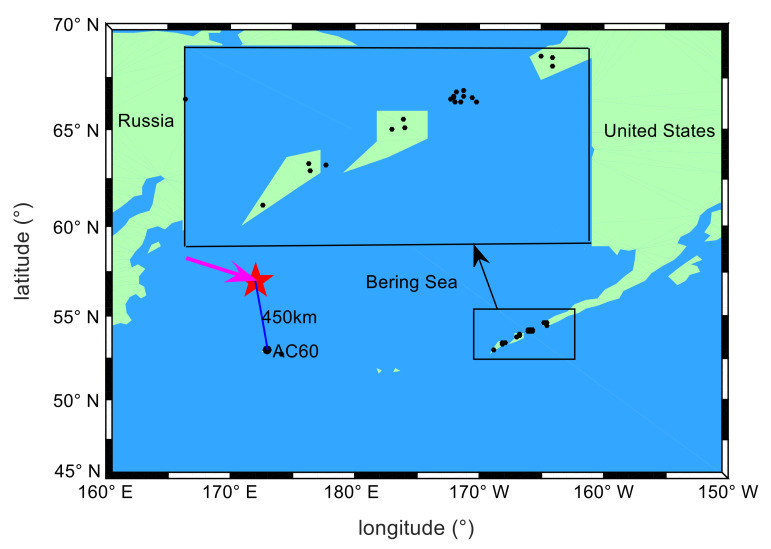
The distribution of the Bering Sea meteor explosion point (red pentagram) and GPS sites (black dots). The purple line with the arrow indicates the trajectory of the meteor movement. The black rectangle is an enlarged view of the distribution of some GPS stations.

Because the Bering Sea meteor explosion occurred at sea, there was only one GPS station (AC60 station) within 900 km of the explosion. The AC60 station is located at 53° N and 173° E, which is about 450 km from the meteor explosion point. During the meteor explosion, the AC60 station could observe six GPS satellites (PRN02, PRN05, PRN07, PRN09, PRN29, PRN30). The observation data from the AC60 station is suitable for analyzing ionospheric disturbances near the meteor explosion. In the western United States, there are 20 GPS stations with an average distance of 1372 km from the meteor explosion. The GPS stations distribution is shown in Figure 1.

**Figure 2:**
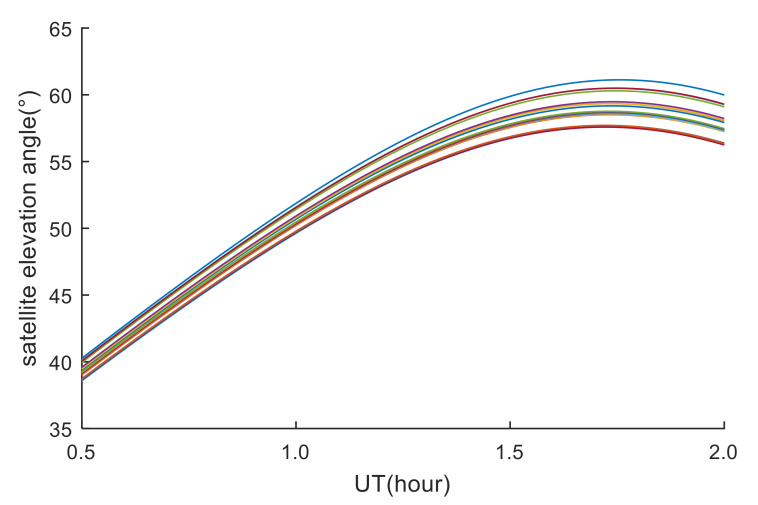
Elevation angle between GPS stations and PRN05 satellite. Lines with different colors represent different GPS stations.

All GPS stations are equipped with dual-frequency receivers. The data sampling rate of three GPS stations is 30 s, and the data sampling rate of other GPS stations is 15 s. The observation data can be obtained from the website https://www.unavco.org/. According to the coordinates of the explosion point and the three-dimensional velocities provided by NASA, the approximate moving trajectory of the meteor is estimated, as shown by the purple arrow in Figure 1. During 00:30:00–02:00:00 UT on the 19th, the observation data of 20 GPS stations and PRN05 satellites passed through the area near the meteor explosion, and the elevation angles of the data are all greater than 37 (the elevation angles of the GPS stations are shown in Figure 2). When the elevation angle is greater than 37 degrees, the GPS data is very little affected by multipath errors. By analyzing the spatial positions of GPS stations and PRN05 satellites, it is found that these GPS stations can be used to detect ionospheric disturbances within the range of 1000–1400 km from the meteor explosion. Therefore, 21 GPS stations can be used to detect ionospheric disturbances caused by the meteor explosion under study.

**Figure 3:**
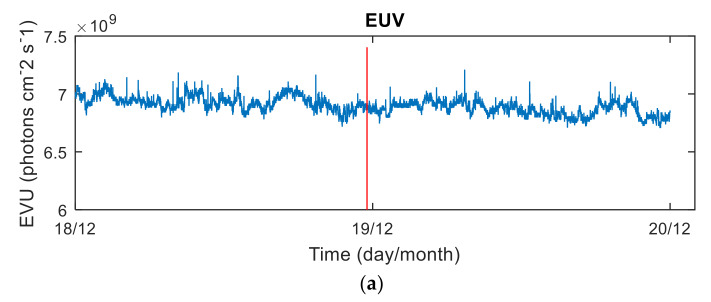

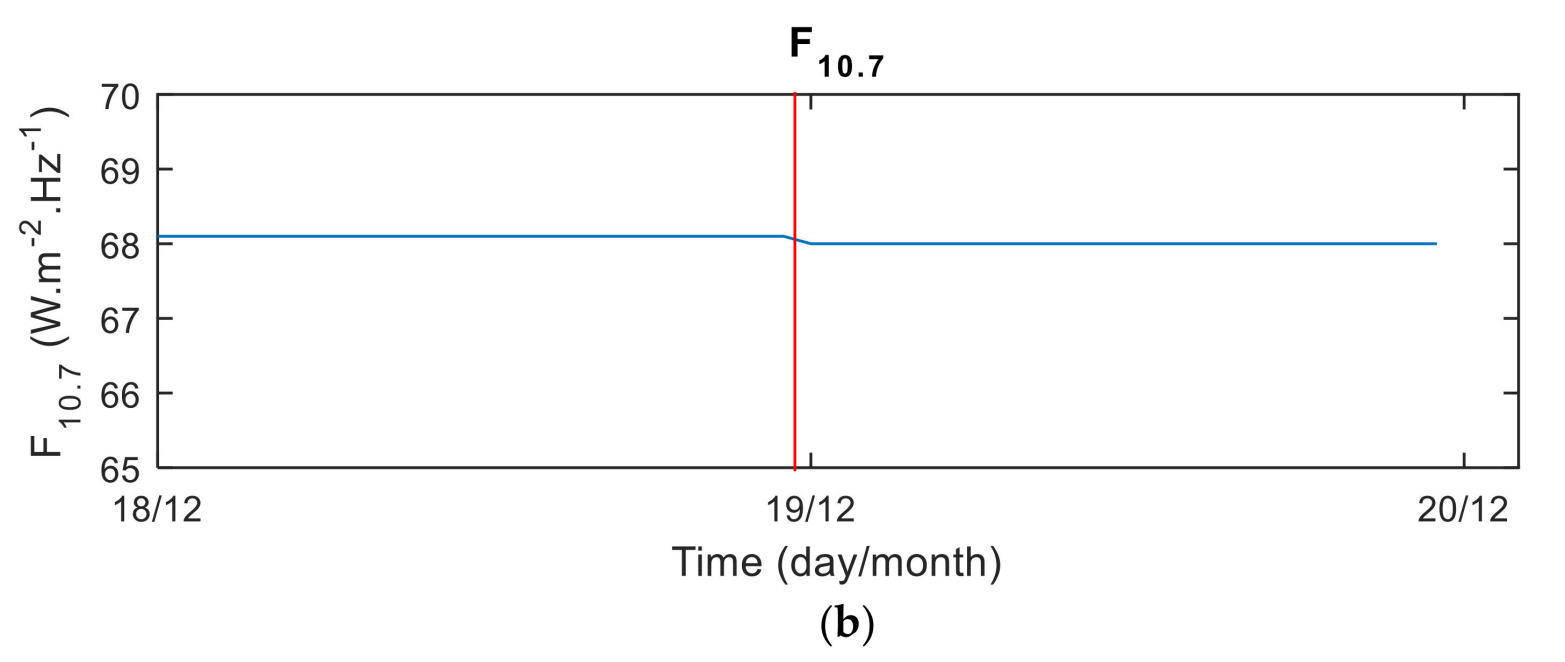
EVU observations (**a**) and F10.7 indicators (**b**) for the period from 18 to 19 December 2018. The red line is the moment of the meteor explosion.

Solar and geomagnetic activities are the main factors that cause ionospheric disturbances. Solar extreme ultraviolet (EUV) radiation is the main ionization source of the Earth’s ionosphere, and its variations change the state of electrons, ions, and neutral particles in the atmosphere. Therefore, EUV observations can be used to describe the intensity of solar activity. The solar radiation level is analyzed using the observation data of 26–34 nm solar EUV radiation provided by the SOHO satellite. The data sampling rate is 15 s. The sun’s 10.7 cm radio radiant flux-F10.7 can be used to indicate changes in the solar EUV radiation (http://omniweb.gsfc.nasa.gov/form/dx1.html). The solar activity is described well by EUV observations and F10.7 [18], as shown in Figure 3. It can be seen from Figure 3 that the solar radiation is stable before and after the meteor explosion and maintains a low radiation level. Therefore, ionospheric disturbance factors due to solar activity can be excluded.

The geomagnetic horizontal component (H component) and vertical component (Z component) from the Japanese geomagnetic observatory Memambetsu (43.91° N, 144° E) are used to analyze the geomagnetic environment. The data-sampling rate is 15 s.

**Figure 4:**
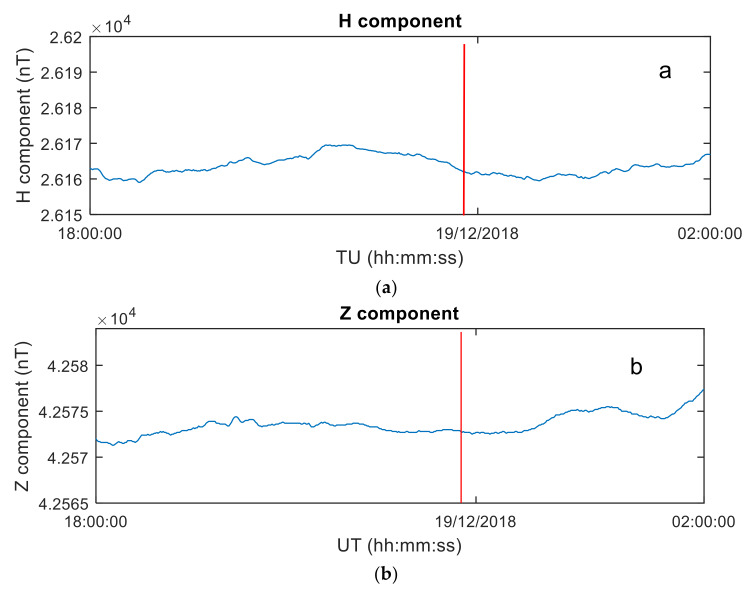
Geomagnetic observation at Memambetsu Station on 18 December 2018, 18:00:00 UT–18:00:00 UT. (**a**) Earth level component (H component) observations. (**b**) the geomagnetic vertical component (Z component). The red line is the moment of the meteor explosion.

It can be seen from Figure 4 that during the explosion, the geomagnetic level near the explosion point is low, and the change is stable, and no obvious geomagnetic fluctuation occurs. At present, KP index and DST index are usually used to describe the geomagnetic conditions. When KP < 5 and DST > −50 nT, the geomagnetic conditions are considered quiet [19]. When the meteor exploded, the geomagnetic condition was very quiet (KP < 1.3, DST > −12). The Kp and Dst data can be obtained from the http://omniweb.gsfc.nasa.gov/form/dx1.html website. Therefore, the possibility of ionospheric disturbances due to geomagnetic activity can be eliminated. This will benefit the analysis the influence of Bering Sea meteor explosion on the ionospheric disturbances.

### 2.2. Data Processing

GPS ionospheric sounding is known to be one of the most powerful tools for remote sensing of the ionosphere. Through the carrier-phase observation data from the GPS station’s dual-frequency receiver, the TEC can be accurately calculated, and the ionospheric disturbance characteristics can be detected by analyzing the TEC variations. Methods of TEC calculation have been described in detail in a number of papers [3,8]. The relative ${\mathrm{TEC}}_{\Phi }(t)$ can be evaluated with high precision and accuracy better than 0.01 TECU from the geometric-free combination of the carrier phase measurements. We reproduce here only the final formula for ${\mathrm{TEC}}_{\Phi }(t)$ [3,20,21]:

(1) $${\mathrm{TEC}}_{\Phi }(t)=\frac{f_{1}^{2}f_{2}^{2}}{40.3(f_{1}^{2}-f_{2}^{2})}(\lambda_{1}\phi_{1}-\lambda_{2}\phi_{2}+\mathrm{const}+\varepsilon )$$

where *f*_1_ and *f*_2_ are the carrier frequencies of GPS (*f*_1_ = 1575.42 MHz and *f*_2_ = 1227.60 MHz). $\phi_{1}$ and $\phi_{2}$ represent the GPS carrier phase measurements, respectively. $\lambda_{1}$ and $\lambda_{2}$ stand for the corresponding wavelengths (*λ*_1_ = 19.03 cm and *λ*_1_ = 24.32 cm). const is an unknown constant deviation. *ε* is residuals. ${\mathrm{TEC}}_{\Phi }(t)$ is the TEC time series. t is the epoch. The Φ in the variable ${\mathrm{TEC}}_{\Phi }(t)$ means TEC calculated using the carrier phase measurement.

As TEC is an integral parameter, it is impossible to determine the height of the TEC disturbance. However, the main contribution to TEC variations would occur around the height of the maximum ionization. This allows us to consider the ionosphere as a thin layer located at height *h*_max_ of the ionosphere F2 layer. In the ionosphere, the height of the maximum electron density is *h*_max_. The intersection of the line of sight (LOS) between each GPS satellite–receiver pair with this layer is an ionospheric pierce point (IPP). The IPP sequences are utilized to track TID’ propagation. Using the data of the GA762 ionosonde station near the meteor explosion point, the average value of the electron density peak height of the F2 layer (hmF2) during the research period was taken as the height of the F2 layer, and then *h*_max_ = 246 km was determined.

Studies show that the power spectrum of the wavelet transform can be applied to better analyze the distribution characteristics of the signal in the time-frequency domain [22,23]. In order to analyze the characteristics of TID in the time-frequency domain, the power spectrum of the TEC time series is calculated based on the wavelet transform. The TEC time series is firstly processed by first-order difference method to eliminate the influence of the linear trend term, and then its power spectrum is estimated. The high energy region in the power spectrum is the ionosphere disturbance. Then the time and period of TID can be determined by analyzing the power spectrum. The power spectrum is defined as follows:

(2) $$E_{a,b}=|W_{f}(a,b){|}^{2}={\left|\left|a{|}^{-\frac{1}{2}}\sum_{i=1}^{N}f(i*\delta t)\psi^{*}(\frac{i*\delta t-b}{a}\right)\right|}^{2}$$

where $E_{a,b}$ is the wavelet power spectrum of the TEC time series. $\psi^{*}(t)$ is the complex conjugate of the mother wavelet ψ(t). Parameters a and b represent the scale and time position of wavelet, respectively. f(t) is the TEC time series after first-order difference processing. δt is the time interval of the signal. N is the length of the signal. $W_{f}(a,b)$ is the wavelet coefficient.

A Butterworth band-pass filter allows us to more easily detect perturbations within an expected range of frequencies [15]. Meteor explosions produce acoustic and gravity waves, which then cause TEC disturbances. Therefore, a Butterworth band-pass filter is applied to isolate acoustic and gravity wave generated TEC disturbances [4,24,25]. When using a Butterworth band-pass filter to analyze the data, the cut-off frequency has strong impacts on the filtering results. Firstly, the cut-off frequency of Butterworth band pass filter is determined according to the power spectrum analysis results of TEC. Then TEC disturbance time series (*DTEC*(*t*)) are obtained by filtering TEC. The abrupt part of TEC disturbance time series is ionospheric disturbance. Finally, the waveform, temporal and spatial distribution, period, propagation velocity and the position of disturbance source of TID caused by meteor explosion are analyzed.

## 3. Analysis of Ionospheric Disturbance Characteristics in the Vicinity of Meteor Explosion at AC60 Station

**Figure 5:**
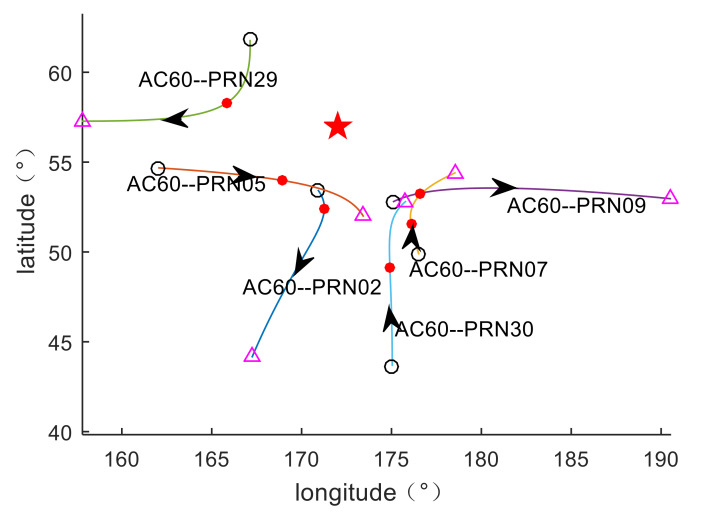
IPP trajectory map. The red pentagram is the position of the explosion point, the black dot is the starting point of the puncture-point track, the triangle is the end point of the puncture-point track, the red dot is the puncture-point position of the meteor explosion, and the arrow indicates the IPP movement direction.

The TEC disturbances are calculated by the method described in Section 2.2, and the corresponding IPP information is estimated. The IPP trajectory distribution between each satellite and receiver is shown in Figure 5. As seen from this figure, at the explosion time of the meteor, the IPP distances of the PRN02, PRN05, PRN07, PRN09, PRN29, and PRN30 satellites are 498, 391, 645, 482, 422 and 884 km, respectively. The IPP of PRN05, PRN07, and PRN30 satellites moved to the explosion point, and PRN05 is closest to the explosion point, which has good TID monitoring conditions. The remaining satellite IPPs move away from the explosion point, but the distance from the explosion point remains within 500 km.

**Figure 6:**
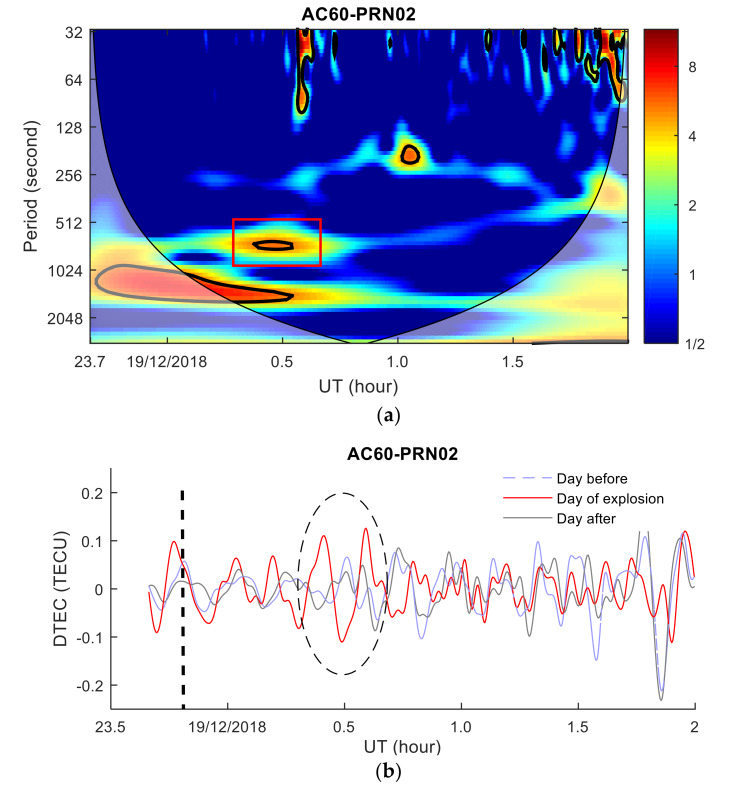

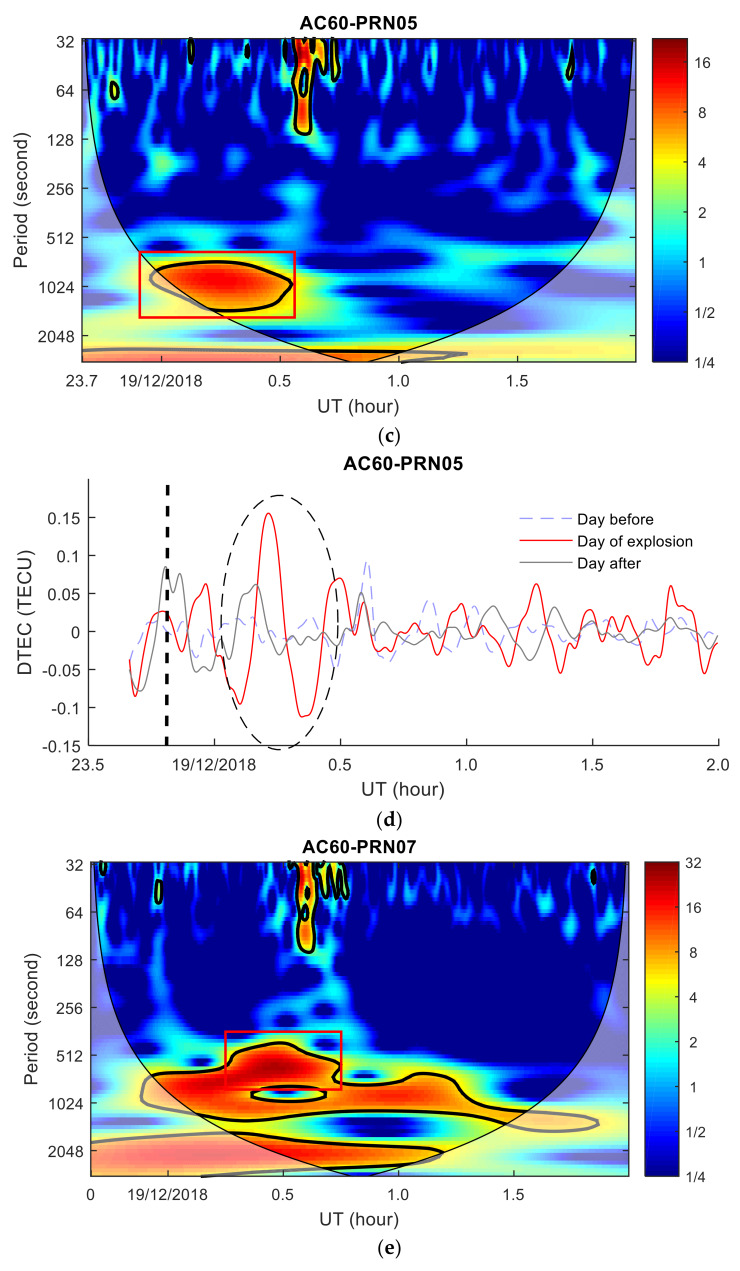

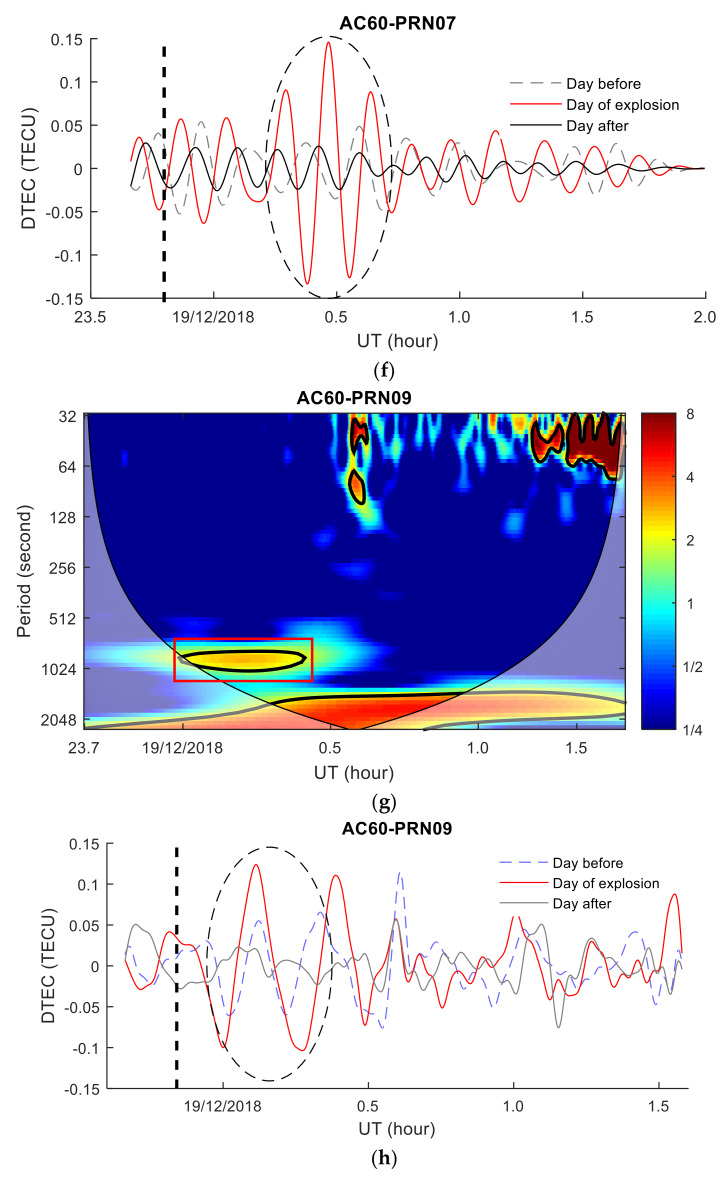
TEC disturbance variations and wavelet transform power spectrum. (**a**,**c**,**e**,**g**) are the power spectra of the TEC time series wavelet transform. The red rectangle is the TEC disturbances area, and the thick black line is the area that passes the 95% confidence test. (**b**,**d**,**f**,**h**) are TEC disturbances variations graphs. The black dotted line indicates the moment of the meteor explosion, the ellipse indicates the disturbances, the blue dotted line indicates *DTEC*(*t*) the day before the explosion, and the black line indicates *DTEC*(*t*) the day after the explosion). Shadow region regions on either end indicate the “cone of influence”, where edge effects become important.

As the elevation angles between AC60 station and PRN29 satellite and PRN30 satellite are small and the observation quality is poor, only the TEC disturbance characteristics of the PRN02, PRN05, PRN07, and PRN09 satellites are used for our analysis. To analyze the TEC disturbance characteristics in the time-frequency domain, the power spectrum of TEC (the PRN02, PRN05, PRN07, and PRN09 satellites) is estimated, as shown in Figure 6a,c,e,g. It can be seen that the TEC disturbances occurred in all four satellites, which are indicated by red rectangles (high-energy regions in the power spectrum). All TEC disturbances detected in time-frequency space have passed a 95% confidence test and are not affected by the boundary effect of the wavelet transform. As Figure 6c shows, the IPP of the PRN05 satellite is closest to the meteor explosion point, and the TEC disturbance is the most significant, with a period of 11–20 min. The TEC disturbance periods of PRN02, PRN07 and PRN09 satellites are 8.5–20 min, and the disturbance intensities are weaker than that of the PRN05 satellite. The cutoff frequency range of the Butterworth filter was determined based on the power spectrum of wavelet transform, and then *DTEC*(*t*) of PRN02, PRN05, PRN07, and PRN09 satellites were calculated using a Butterworth filter. To verify that the TEC disturbance during this period is caused by the meteor explosion, the TEC disturbances the day before and after the meteor explosion are calculated and compared. The *DTEC*(*t*) disturbances are shown in Figure 6b,d,g,h. It can be seen that all four satellites show obvious TID; the disturbances range from 0.11 to 0.15 TECU, and the appearance time of the TID are consistent with the TID detected by the time-frequency maps. The TEC disturbance exhibits a distinct N-shaped shock wavefront feature, which is remarkably similar to the ionospheric disturbance characteristics induced by explosions near the ground [13], with the typical wavefront characteristics of a shock wave. The TEC disturbance waveforms of the four satellites are different. The disturbance waveforms are divided into three categories. The first type of waveform has a maximum value and a minimum value; the second type has two maximum values and one minimum value; and the third type has multiple maximum and minimum values. PRN05 shows the first type; PRN02 shows the second type; and PRN07, PRN09 show the third type of waveform alone or a combination with the first and second types. The first type of waveform appears closer to the explosion point (the distance is 380.86 km), and the third type of waveform appears at a long distance, which is consistent with the conclusion drawn by Perevalova et al. [16]. There was no similar TEC disturbances in the same time period on the day before and after the meteor explosion. As the low levels and small changes in geomagnetic and solar activity before and after the meteor explosion, the TEC disturbances detected by the four satellites are probably caused by the meteor explosion.

**Figure 7:**
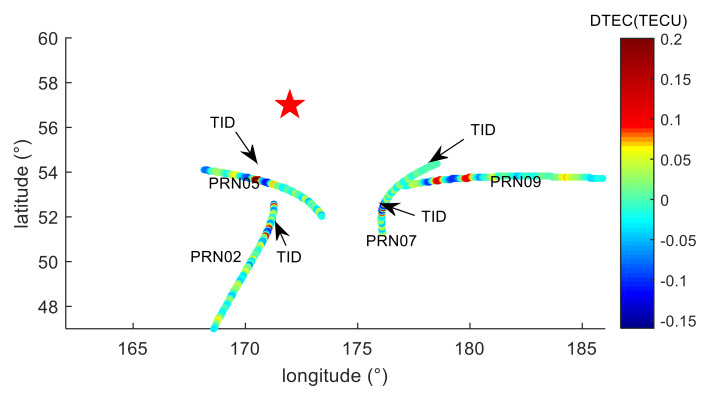
Spatial distribution of the TEC disturbance. The black arrow in the figure points to the TID position. The red five-pointed star is the location of the meteor explosion.

To further analyze the spatial distribution characteristics of ionospheric disturbance near the meteor explosion, the TEC disturbances distribution was shown in Figure 7. The black arrow in Figure 7 indicates where the TID appears. Due to the distribution of the visible satellites at the AC60 station, the IPP trajectories are mainly distributed to the south of the meteor explosion, and TID was detected in the area about 380–600 km south of the meteor explosion point. The spherical distances from the TID detected by the four satellites to the meteor explosion point are calculated, respectively, and then the distances are divided by the TID propagation time to estimate the average horizontal propagation velocity of TID. The spatial position where the TID appear, the distances between the TID and the explosion point, the azimuth angle formed by the TID and explosion point, the elevation angle, the average horizontal propagation velocity of the TID and the magnitude of the TID are counted and shown in Table 1. The propagation velocities of TID detected by PRN02, PRN05 and PRN07 satellites are similar, and the velocities are about 238.13–270.94 m/s. The propagation velocity detected by the PRN09 satellite is about 434.02 m/s. The direction and elevation angle between the receivers and the satellites, the distances between TID and the explosion point, and the azimuth angle of the TID in the AC60 station are different, so the magnitude of the detected TID and propagation velocities are different, indicating that the TID generated by the meteor explosion is anisotropic, and the TID characteristics are related to the spatial distribution, which is consistent with the results of [13,16].

## 4. The Analysis of Long-Distance TID Characteristics Based on GPS Observations

**Figure 8:**
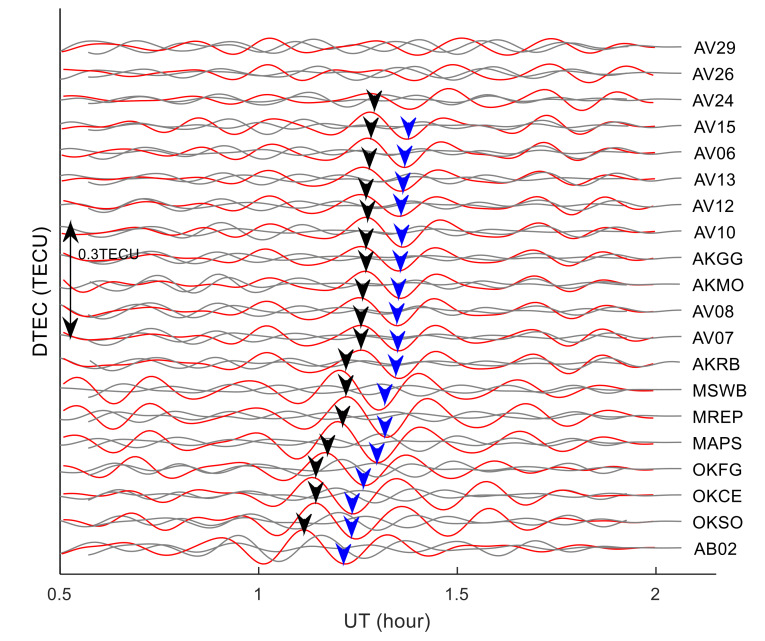
Time series of TEC disturbance between 20 GPS stations and PRN05. The red curve is the *DTEC*(*t*) on 19th, the gray curve is the *DTEC*(*t*) on 18th and 20th, the black arrow indicates the maximum amplitude position of TID, and the blue arrow indicates the minimum amplitude position of TID.

The TEC observations from 20 GPS stations within the range of 1000–1450 km from the meteor explosion point and PRN05 satellite were used to analyze the propagation characteristics of long-distance TID. The distribution of GPS stations is shown in the enlarged diagram in Figure 1. The distances from the GPS stations to the meteor explosion point are sorted. The nearest GPS station is AB02, and the farthest station is AV29. The distances order from near to far is shown in Figure 8. The TEC disturbance time series (*DTEC*(*t*)) of 20 GPS stations and PRN05 are calculated respectively using the method introduced in Section 2.2. TID were found in 17 stations and the TID of each station is indicated by the arrows in Figure 8. To further verify that the TID were caused by the meteor explosion, the *DTEC*(*t*) of the same time period on the 18th and 20th were calculated (as shown by the gray curve in Figure 8). It can be seen from Figure 8 that TID were detected from all from AB02 station to AV15 station. The time duration of TID increases as the distances between the GPS stations and the meteor explosion point increase, and the TEC disturbance amplitudes decrease with the increasing distances, which implies severe dissipation of the perturbation amplitude during the propagation of the TID. No obvious TID was found in AV24, AV26 and AV29 stations, which indicates that the TID did not reach the observational range of them. No similar TEC disturbances were found on the *DTEC*(*t*) curves of the 20 GPS stations during the same period of the 18th and 20th. During this period, the solar radiation and geomagnetic environment are stable, and there are no large-scale earthquakes, tsunamis and other TEC disturbance sources near the meteor explosion point. Therefore, the TID detected during this period on the 19th is most likely caused by the meteor explosion.

**Figure 9:**
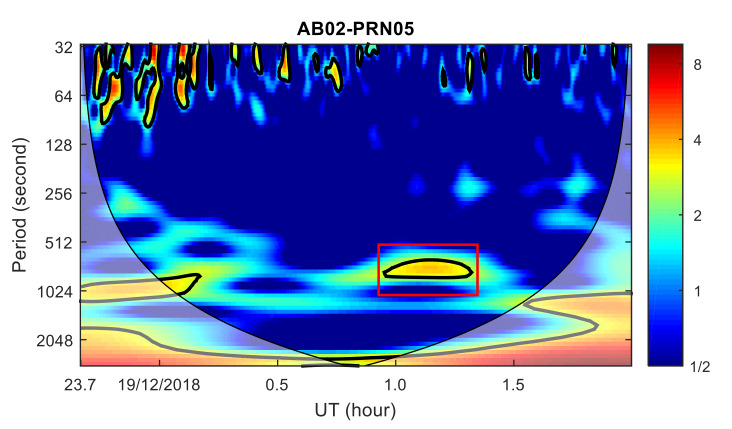
Wavelet transform power spectrum of TEC time series at AB02 station. Shadow region regions on either end indicate the “cone of influence”, where edge effects become important.

**Figure 10:**
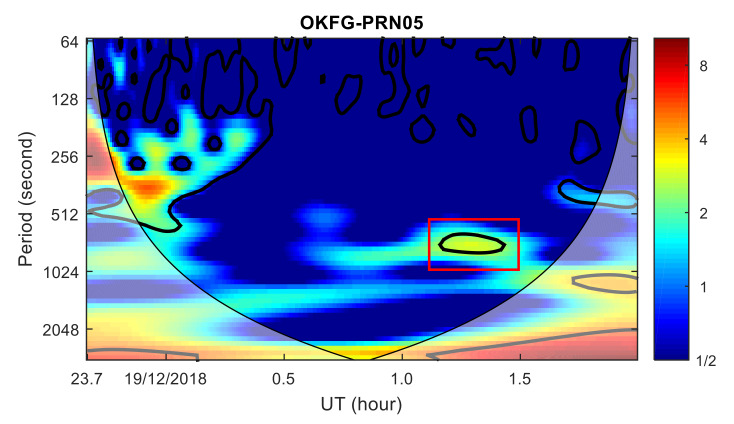
Wavelet transform power spectrum of TEC time series at OKFG station. Shadow region regions on either end indicate the “cone of influence”, where edge effects become important.

**Figure 11:**
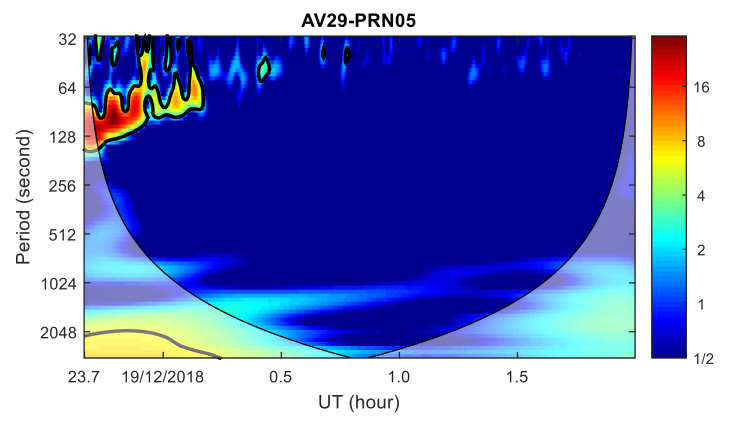
Wavelet transform power spectrum of TEC time series at AV29 station. Shadow region regions on either end indicate the “cone of influence”, where edge effects become important.

To analyze the ionospheric disturbance characteristics from the frequency domain, the wavelet transform power spectrum of the TEC time series from AB02, OKFG and AV29 stations with PRN05 satellite were calculated, respectively, as shown in Figure 9, Figure 10 and Figure 11. TID was found in the wavelet transform power spectrum of the AB02 and OKFG stations, with a period of about 9–16 min, which is within the period range of AC60 station. As the distance between the OKFG station and the meteor explosion point is greater than that of the AB02 station, the TID intensity of the AB02 station is greater than that of the OKFG station, while the TID of the OKFG station appears later than that of the AB02 station. No obvious TID was found in the wavelet transform power spectrum at the AV29 station. The TID propagation characteristics found in the time domain was further verified by the TID propagation characteristics found in the frequency domain. The propagation velocities and periods of the TID are consistent with the propagation characteristics of the TID discovered by the AC60 station, which means that these TIDs are caused by the same disturbance source and are related to the meteor explosion. Since 17 GPS stations are further away from the meteor explosion point than the AC60 station, the disturbance amplitudes of TID found in these stations is much smaller than that of AC60 station, which implies severe dissipation of the perturbation amplitude during the propagation of the wave.

**Figure 12:**
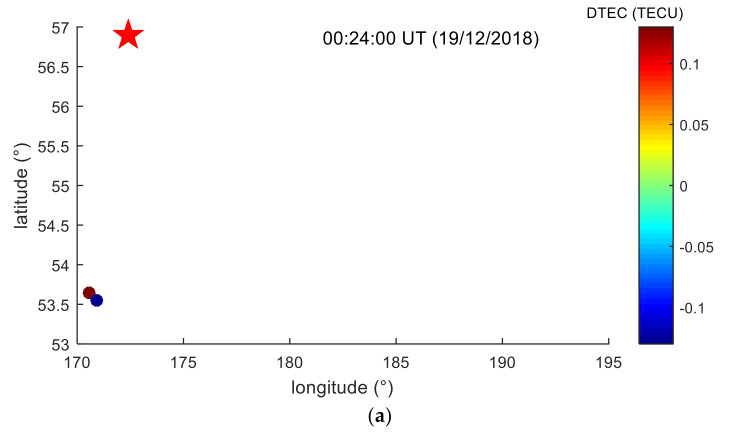

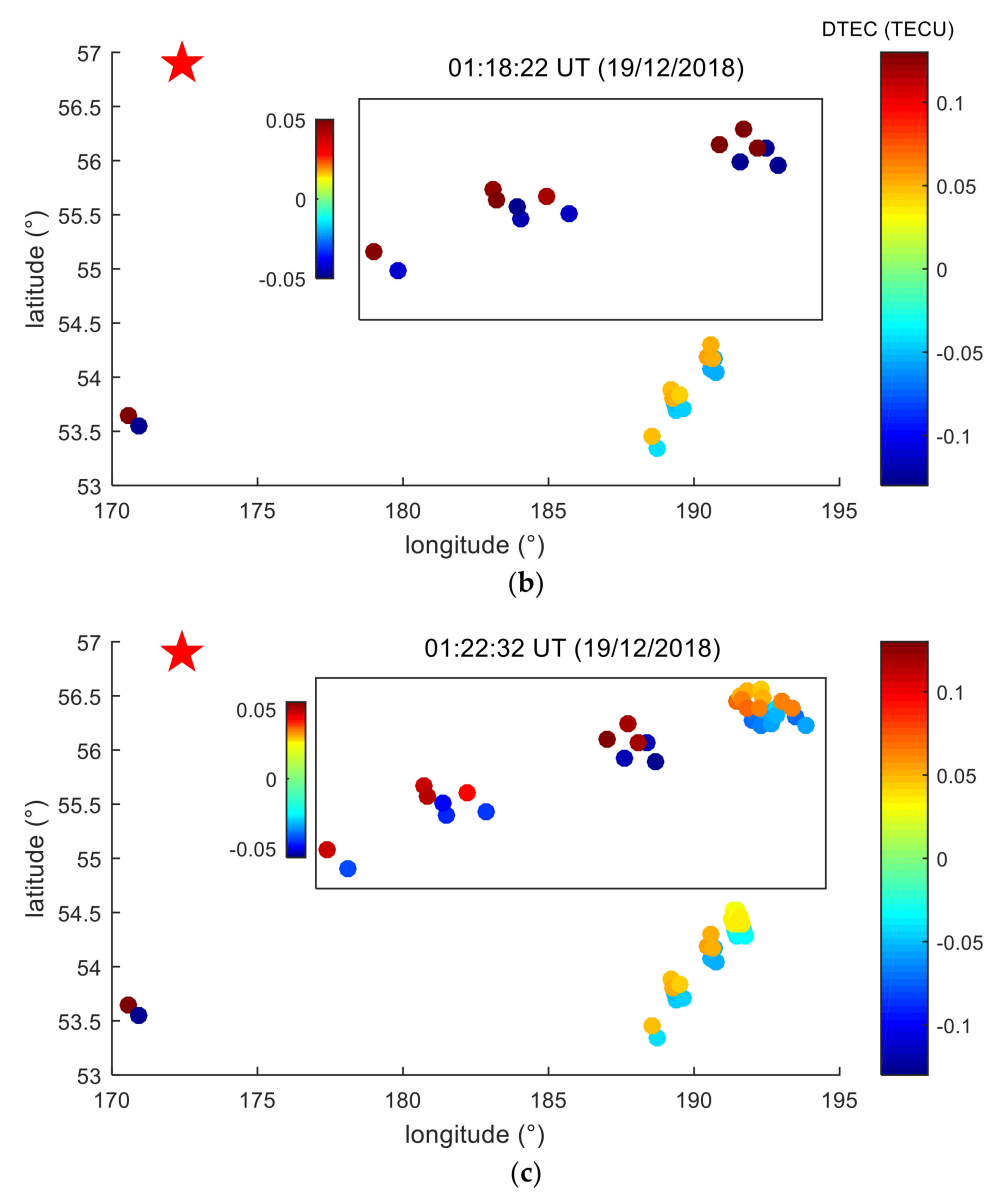
The spatial distribution of the maximum and minimum TEC of TID at different times. The five-pointed star is the location of the meteor explosion point. The rectangular frame is an enlarged view of the detected TID in 18 GPS stations. (**a**) is the spatial distribution of TID detected before 00:24:00 UT. (**b**) is the spatial distribution of TID detected before 01:18:22 UT. (**c**) is the spatial distribution of TID detected before 01:22:32 UT.

In order to be able to intuitively verify that the TID is caused by a meteor explosion and to understand the dynamic propagation process of TID in two-dimensional space, the maximum and minimum TEC spatial distributions of TID in different time regions are drawn, as shown in Figure 12. Figure 12 shows that all the TIDs are located to the southeast of the meteor explosion, because the GPS station is located in the southeast of the explosion point. By analyzing the dynamic change of TID position, the propagation process of TID in the southeast direction of the meteor explosion can be well analyzed. At 00:24:00 UT, the first TID was found at about 380.86 km from the meteor explosion point, as shown in Figure 12a. In the next 54 min (Figure 12b), seven new TIDs were detected in the southeast, which indicated that the TEC disturbance propagated southeast to 1100–1200 km from the meteor explosion. During the period of 01:18:22–01:22:32 UT (Figure 12c), TEC disturbance continued to propagate southeast, and there were 10 new TIDs in the southeast. Since no new TID was found after 01:22:32 UT, the new TID distribution map was not continued. Figure 12 shows that TEC disturbance propagates approximately radially in the southeast of meteor explosion, and stops propagating after 01:22:32 UT, or the amplitude of TID is very weak. The amplitude of the TID weakens with increasing distance from the meteor explosion, indicating that the TEC disturbance consumes energy during the propagation process, and finally results in no new TID after 01:22:32 UT. The temporal and spatial distribution characteristics of the TID found in Figure 12 are similar to the TEC disturbance caused by acoustic waves and gravity waves.

## 5. Estimation of TID Propagation Velocity and Disturbance Source Location

**Figure 13:**
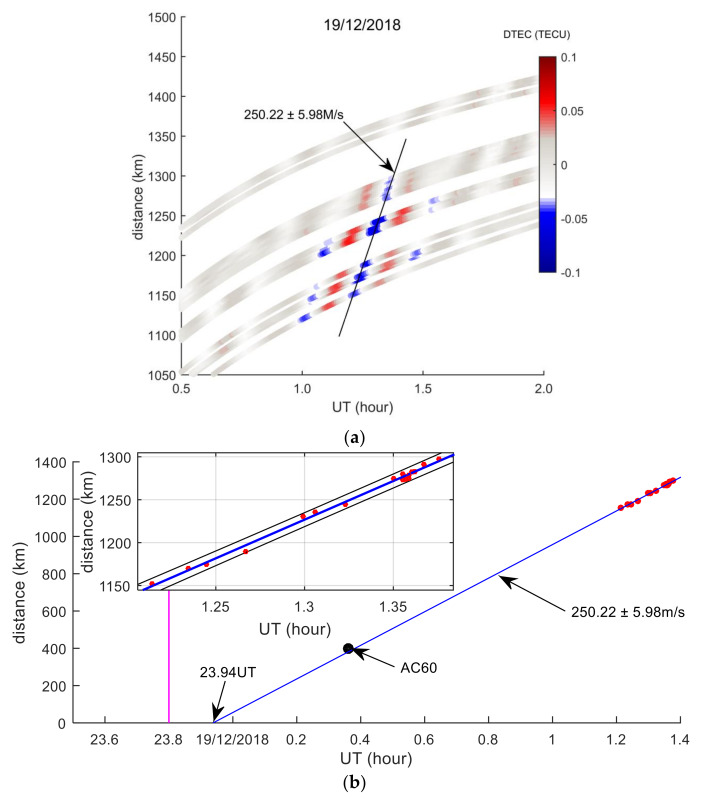
TEC disturbances distance-time graph estimated by 20 GPS stations and PRN05 satellite on 19th. The abscissa represents time, and the ordinate represents the spherical distances between IPPs and the meteor explosion point. The color in (**a**) represents the TEC disturbance time series (*DTEC*(*t*)). The slope of the line indicates the velocity of TID propagation in (**b**). The rectangular frame is an enlarged view of the TID, and the black dotted line indicates the 95% confidence interval. The black dot is the TID of the AC60 station, and the purple line indicates the time of the meteor explosion.

**Figure 14:**
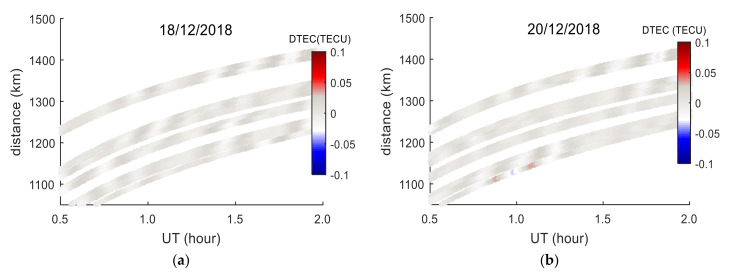
TEC disturbance distance-time graphs estimated by 20 GPS stations and PRN05 satellites on 18th and 20th.

To accurately estimate the propagation velocity of TID, the *DTEC*(*t*) of each GPS station and the coordinates of corresponding IPPs are estimated. The coordinates can be used to calculate the spherical distances between IPPs and the meteor explosion point, as shown in Equation (3). Then the distance-time diagram of TEC disturbances was drawn based on *t*, distance and *DTEC*(*t*), as exhibited in Figure 13. A TID propagation mode can be found in Figure 13a, that TID propagates away from the meteor explosion point (radial propagation), and the magnitude of TID decreases with increasing distance. The TID in Figure 13a was linearly fitted, and the fitting result is shown in Figure 13b. The slope of the blue line is the velocity of TID propagation, and the average propagation velocity of TID is determined as 250.22 ± 5.98 m/s. It can be seen from Figure 13b that the blue line intersects the time axis at 23.94 UT (about 8 min after the meteor explosion), indicating that an ionospheric disturbance source formed 8 min after the meteor explosion. The TID detected by the AC60 station (black dot in Figure 13b) is very close to the blue line, indicating that the TID detected by the AC60 station has the same propagation characteristics as the TID in Figure 13a, and these TIDs are caused by the same TEC disturbance source. In order to verify that the detected TID is related to the meteor explosion, the TEC disturbance maps were drawn on 18 and 20 December, respectively, as shown in Figure 14. Figure 14 shows that there was no obvious ionospheric disturbance on 18 and 20 December. During the meteor explosion, the geomagnetic and solar radiation conditions are very quiet, and there are no earthquakes, tsunamis and other events near the meteor explosion. Therefore, it is verified that the detected TID is caused by the meteor explosion:

(3) $${\mathrm{distance}}_{i}=(R+h_{\max})\arccos(\sin(B_{0})\sin(B_{i})+\cos(B_{0})\cos(B_{i})\cos(L_{i}-L_{0}))$$

where, *R* is the radius of the Earth (6371 km), *h*_max_ = 246 km. (*B_i_*, *L_i_*) is the IPP position corresponding to the largest TEC of the TID. *i* is the number of the GPS station.

**Figure 15:**
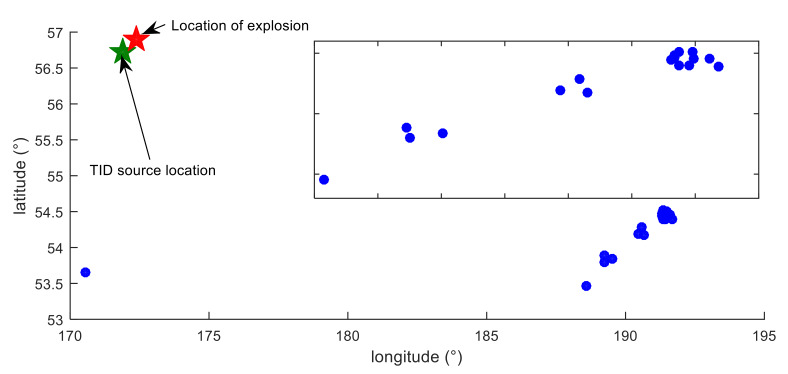
Estimation of TID source location. The red pentagram is the meteor explosion point, the green pentagram is the estimated TID source location, the blue dot is the position of the maximum amplitude of the TID, and the rectangular frame is an enlarged view of part of the TID.

The observation equation is established by using the IPP positions and propagation time corresponding to the largest TEC of the TID, then the position and propagation velocity of the TID source can be solved by the least squares method. Observation equation is shown in Equation (4), then Equation (4) is linearized, and (*B*_0_, *L*_0_) and v can be solved. The optimization toolbox provided by Matlab can also be used to solve the Equation (4). The estimated location of the TID source (56.78 ± 0.10°, 171.90 ± 0.36°) is close to the location of the meteor explosion, with a deviation of 39.72 km. The location of the TID source and the location of the meteor explosion point are shown in Figure 15. The estimated TID propagation velocity (v=256.12±5.42 m/s) is close to that in Figure 13. The location deviation between the meteor explosion and the estimated TID source is mainly caused by three factors. First, TEC and IPP positions estimation errors; second, the energy dissipation of TID during long-distance propagation; third, the anisotropy of meteor explosion disturbances. The estimated propagation velocity of TID is in accordance with the propagation velocity of acoustic gravity waves in the ionosphere:

(4) $$t_{i}=(R+h_{\max})\arccos(\sin(B_{0})\sin(B_{i})+\cos(B_{0})\cos(B_{i})\cos(L_{i}-L_{0}))/v-t_{0}$$

where (*B*_0_, *L*_0_) is the position of the TID source to be estimated. (*B_i_*, *L_i_*) is the IPP position corresponding to the Minimum TEC of the TID. v is the propagation velocity to be estimated of the TID. $t_{0}=0.06$ is determined according to Figure 13 and *t_i_* is the time corresponding to the TID.

## 6. Discussions

The AC60 station was used to analyze the ionospheric response to the Bering Sea meteor explosion in the range of 380–600 km. As seen from the TEC series (Figure 6b,d,f,h), the disturbance was characterized by an “N”-shaped shock front with a maximum amplitude of 0.13–0.16 TECU. The first type of TEC disturbance waveform appeared in the area closer to the explosion point (380.86 km). The third type of waveform appeared in a relatively far area, as shown in Figure 8 (distance greater than 1000 km). A typical N-type TEC disturbance with an amplitude of 0.07–0.6 TECU was also triggered by the Chelyabinsk meteorite blast event [12,14,15,16]. Similar TEC disturbance characteristics also were found in earthquakes, rocket launches, nuclear explosions, and tsunamis [3,4,5,6,7]. Due to the direction, elevation angle between the receiver of the AC60 station and the satellite, the distances between the TID and the explosion point, and different azimuth angle of the TID, the detected magnitude of the TID and the propagation velocities are different, which indicating that the TID caused by the meteor explosion is anisotropic and the result is consistent with Yuri’s research [13].

Two types of TID propagation modes were discovered. The propagation velocity of the first type of TID is 238.13–270.94 m/s, and that of the second type of TID is ~434.02 m/s. A few TID velocity modes ranging approximately from 250 to 660 m/s were distinguished from the distance-time diagram analysis, which are generally consistent with early estimations [9,17], and the recent determinations [15]. These two types of TID belong to MSTID from the perspective of wavelength and frequency. Their velocities overlap the propagation velocity of gravity waves in the ionosphere (approximately 200 to 800 m/s), together with the similar period, suggests that this type of TID was excited by a gravity wave triggered by the meteor explosion. The similar TID propagation velocity and period are discovered in the Chelyabinsk meteorite blast, nuclear explosions and tsunamis, confirming that this type of TID is excited by a gravity wave.

The spatial and temporal characteristics of TID within 1000–1400 km southeast of the meteor explosion point were analyzed based on the GPS observations. It can be seen from Figure 8 that, the TEC disturbance amplitude decreases as the distance between the GPS station and the meteor explosion point increases. No TID is detected at AV24, AV26, and AV29 stations, which implies the disturbance amplitude is severely dissipated during wave propagation. No similar TID was found on the 18th and 20th, indicating that the TID is most likely related to the meteor explosion. Through spectrum analysis of the TEC time series, it is found that the periods of 17 TID are about 9–16 min, and the average period is about 12 min. TID approximately propagates radially. If there were more GPS stations near the meteor explosion point, the characteristics of TID radial propagation will be more prominent, and maybe more kinds of TIDs will be found.

By analyzing the occurrence time and propagation distance of the TID detected by 17 GPS stations (Figure 12 and Figure 13), the average horizontal propagation velocity of TID is estimated to be 250.22 ± 5.98 m/s, the wavelength is about 135–240 km, the average period is about 12 min, and the propagation distance is less than 1400 km. As seen in Figure 13b, the TID detected by the AC60 station and the PRN05 satellite are close to the blue straight line, and the periods of these TID are similar, which is likely to be the same TEC disturbance mode. The detected TID propagation velocities, periods and wavelengths conform to the characteristics of gravity waves propagation in the ionosphere, which belongs to MSTID [26,27]. A similar TID propagation velocity was excited by the Chelyabinsk meteorite blast event [12,14,28], and this type of TID was determined to be caused by a gravity wave triggered by the meteor explosion. This kind of low velocity TID propagation distance generally does not exceed 1500 km. In terms of the velocity, period and distance of TID propagation, the characteristics of the Bering Sea meteor explosion TID are very consistent with Chelyabinsk meteorite blast. The results of these studies further verified that the meteor explosion produced gravity waves, and the propagation of gravity waves in the ionosphere triggered such low velocity TID. Similar low velocity TID have been observed in earthquakes, nuclear explosions, and tsunami events [3,4,5].

**Figure 16:**
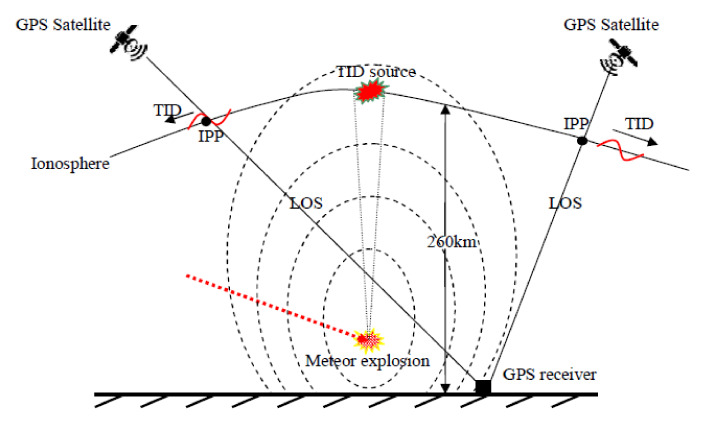
Meteor explosion induced ionospheric disturbance model.

The formation time and location of the TID source are determined using the GPS network, which are very consistent with the location and appearance time of the meteor explosion. At 23.94 UT (about 8 min after the meteor explosion) on the 18th, a disturbance source of TID was formed in the F2 layer above the meteor explosion point. Ionospheric disturbance caused by meteor explosion can be seen in Figure 16. High velocity meteor explosion generated powerful shock waves while acoustic perturbations in the atmosphere led to the upward propagation of acoustic and gravity waves into the ionosphere, then at 23.94 UT, there formed a TID source in the ionosphere, and the TID propagates in the ionosphere. When line of sight (LOS) of GPS receiver and satellite pass through the ionospheric disturbance zone, TID can be observed by GPS net. Two types of TID (~250 m/s, ~434 m/s) propagation modes are detected. It takes about 8 min for the gravity waves generated by the meteor explosion to propagate upward to the ionosphere and form the TID source. The average velocity of the gravity wave propagating upward is 464.58 m/s (*h*_max_ divided by 8 min), which is consistent with the propagation characteristics of the gravity wave in the atmosphere. Waveforms typical for TEC disturbances induced by the gravity waves generated from earthquakes, explosions, and rocket launches were identified. The findings in these studies can further verify and explain the ionospheric response caused by a meteor explosion. The location of the TID source was estimated, and it coincides with the location of the meteor explosion point, which further verifies that the detected TID is caused by the meteor explosion. The location deviation between the meteor explosion and the estimated TID source is mainly caused by three factors. First, TEC and IPP positions estimation errors; second, the energy dissipation of TID during long-distance propagation; third, the anisotropy of meteor explosion disturbances.

## 7. Conclusions

In this paper, with GPS observations, the significant ionospheric disturbances were detected and the waveforms, spatiotemporal distribution, periods, propagation velocities and the disturbance sources of TID were also analyzed. The analysis results verified that the detected TID was caused by the Bering Sea meteor explosion and accounted for the formation principle and propagation characteristics of the TID. The results are of great significance to further study the ionosphere’s response to meteor explosions.

The TID was detected in both time and frequency domains by means of Butterworth band-pass filter and wavelet transform method, and the occurrence time of TID they detected is consistent. The waveform of TID has obvious “N-shaped” characteristics and can be divided into three types. It is found that TID is anisotropic.

Two types of TIDs were found in the vicinity of the meteor explosion point. The propagation velocity of the first type of TID was 238.13–270.94 m/s, and the second type ~434.02 m/s. Within 1000–1400 km southeast of the meteor explosion point, the TID is detected of which the horizontal propagation velocity is 250.22 ± 5.98 m/s, the wavelength is about 135–240 km, and the average period is about 12 min. The TID propagation distance is less than 1400 km. By analyzing the wavelengths, periods and velocities of TID, the TID detected in this area is considered as the first type. The TID caused by gravity waves excited by the meteor explosion conforms to the characteristics of gravity waves propagation in the ionosphere, which was confirmed to MSTID.

The formation and propagation of TID caused by meteor explosion are explained in detail. After the meteor explosion, a gravity wave is generated and propagates upward at a velocity of 464.58 m/s, and it reached the F2 layer about 8 min. A TID disturbance source is formed in the F2 layer above the meteor explosion point and then the TID propagates radially in the ionosphere. The location of the TID source is estimated as 56.78 ± 0.10°, 171.90 ± 0.36° using the spatiotemporal data of TID. The estimated TID source location is close to the location of the meteor explosion, with a deviation of 39.72 km. This deviation is reasonable due to the anisotropy of TID propagation and the error of TID spatiotemporal data. The location of the TID source coincides with the meteor explosion, which well verifies that the TID is caused by the meteor explosion.

## Figures and Tables

**Table 1:** TID spatial distribution characteristic parameters.

Track Number	IPP Location	Distance between TID and Explosion Point	Elevation Angle	Azimuth Angle	Disturbance Magnitude	Average Horizontal Propagation Velocity
AC60-PRN02	51.61° N/ 171.10° E	595.79 km	44.12	188.70	0.13 TECU	270.94 m/s
AC60-PRN05	53.66° N/ 170.50° E	380.86 km	43.82	199.30	0.16 TECU	248.33 m/s
AC60-PRN07	52.42° N/ 176.13° E	575.27 km	60.08	152.70	0.14 TECU	238.13 m/s
AC60-PRN09	53.62° N/ 178.50° E	532.47 km	45.53	130.80	0.13 TECU	434.02 m/s
